# Neurohumoral Control of Sinoatrial Node Activity and Heart Rate: Insight From Experimental Models and Findings From Humans

**DOI:** 10.3389/fphys.2020.00170

**Published:** 2020-03-03

**Authors:** Eilidh A. MacDonald, Robert A. Rose, T. Alexander Quinn

**Affiliations:** ^1^Department of Physiology and Biophysics, Dalhousie University, Halifax, NS, Canada; ^2^Cumming School of Medicine, Libin Cardiovascular Institute of Alberta, University of Calgary, Calgary, AB, Canada; ^3^School of Biomedical Engineering, Dalhousie University, Halifax, NS, Canada

**Keywords:** acetylcholine, adenosine, catecholamines, G-protein-coupled receptors, heart rate, intracellular signaling, natriuretic peptides, regulation

## Abstract

The sinoatrial node is perhaps one of the most important tissues in the entire body: it is the natural pacemaker of the heart, making it responsible for initiating each-and-every normal heartbeat. As such, its activity is heavily controlled, allowing heart rate to rapidly adapt to changes in physiological demand. Control of sinoatrial node activity, however, is complex, occurring through the autonomic nervous system and various circulating and locally released factors. In this review we discuss the coupled-clock pacemaker system and how its manipulation by neurohumoral signaling alters heart rate, considering the multitude of canonical and non-canonical agents that are known to modulate sinoatrial node activity. For each, we discuss the principal receptors involved and known intracellular signaling and protein targets, highlighting gaps in our knowledge and understanding from experimental models and human studies that represent areas for future research.

## Introduction

The heart’s natural pacemaker, the sinoatrial node (SAN), spontaneously initiates each heartbeat. Located adjacent to the *crista terminalis* at the junction of the right atrium and the superior *vena cava*, the SAN fires action potentials (AP) that propagate through the electrical conduction system and the myocardium, initiating contraction. This specialized group of cells is necessary for life, as its rhythmical firing leads to the pumping of blood to the rest of the body. Because its function is so essential, the SAN has many redundant systems in place to ensure it continues to consistently generate APs (Difrancesco and Noble, 2012; Maltsev and Lakatta, 2012; Rosen et al., 2012). The frequency of AP firing from the SAN determines heart rate (HR), and there are a multitude of factors that influence the systems that generate the SAN AP. Thus, SAN activity is tightly controlled to maintain normal heart rhythm and allow for adaption to changes in physiological demand.

**FIGURE 1:**
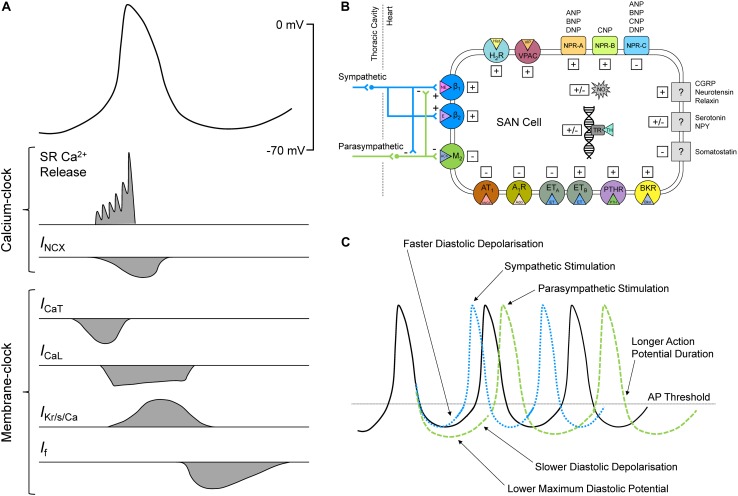
Sinoatrial node (SAN) cell action potential (AP) and ionic fluxes responsible for automaticity **(A)**, neurotransmitters and neuropeptides, autocrine, paracrine, and endocrine factors, and other biologically active agents with known effects on SAN activity and heart rate **(B)**, effect of sympathetic and parasympathetic stimulation on the SAN AP **(C)**. A_1_R, adenosine receptor 1; ACh, acetylcholine; Ado, adenosine; ANGII, angiotensin II; ANP, atrial natriuretic peptide; AT_1_, angiotensin receptor 1; BNP, brain natriuretic peptide; Bkn, bradykinin; BKR, bradykinin receptor; Ca^2+^, calcium; CGRP, calcitonin gene-related peptide; CNP, C-type natriuretic peptide; DNP, *Dendroaspis* natriuretic peptide; E, epinephrine; ET, endothelin; ET_A/B_, endothelin receptor A/B; H_2_R, histamine H2 receptor; Hist, histamine; *I*_CaL_, long-lasting (L-type) Ca^2+^ current; *I*_CaT_, transient (T-type) Ca^2+^ current; *I*_f_, “funny” current; *I*_Kr/s__/__Ca_, rapid delayed rectifier / slow delayed rectifier / Ca^2+^-activated K^+^ currents; *I*_NCX_, Na^+^-Ca^2+^ exchanger current; M2, muscarinic receptor 2; NE, norepinephrine; NO, nitric oxide; NPR, natriuretic peptide receptor; NPY, neuropeptide Y; PTH, parathyroid hormone; PTHR, PTH receptor; SR, sarcoplasmic reticulum; TH, thyroid hormone; TR, thyroid receptor; VIP, vasoactive intestinal polypeptide; VPAC, vasoactive intestinal polypeptide receptor.

Modulation of SAN activity occurs through intracellular signaling and its alteration by neurohumoral agents, including neurotransmitters and neuropeptides, and autocrine, paracrine, and endocrine factors (Irisawa et al., 1993; MacDonald et al., 2017), as well as response to changes in mechanical load (Quinn and Kohl, 2012). Neurohumoral agents primarily (although not exclusively) activate a variety of G-protein coupled receptors (GPCR) in the sarcolemma of SAN myocytes (Marin-Garcia, 2011). GPCR in turn activate second messenger cascades that target intracellular components responsible for SAN automaticity, causing a change in HR (Mangoni and Nargeot, 2008; Figure 1). Many of the factors that modulate SAN activity have been extensively studied and are understood to be principally important for the control of HR. Other factors, especially those with secondary effects on the SAN, are less well understood, complicated by contradictory results in the literature. A list of neurohumoral agents, their receptors, and their effect on HR is found in Table 1.

**TABLE 1:** Summary of neurohumoral agents with known effects on heart rate (HR).

**Neurohumoral agent**	**Type**	**Receptor(s)**	**HR effect**	**Species**	**Pathway(s)**	**References**
Norepinephrine (NE) / Epinephrine (E)	Neurotransmitters and circulating hormones	β_1_	↑		G_s_	Drouin, 1997
		β_2_	↑ or ↓		G_s_ and G_i_	
		β_3_	variable		G_s_ and G_i_	
		α_1_	?		G_q_/11	
Acetylcholine (ACh)	Neurotransmitter	M_2_	↓		G_i_ and *I*_KACh_ activation	Drouin, 1997
Histamine	Neurotransmitter and hormone	H_1_	↑		G_i_	Matsuda et al., 2004
		H_2_		Human, rabbit, rat, guinea pig, python	G_s_	Satoh, 1993; Hattori et al., 2004; Matsuda et al., 2004
		H_3_ and H_4_	none	Guinea pig, human	G_i_	Levi and Smith, 2000
Serotonin (5-HT)	Neurotransmitter	5-HT_3_	↓	Rat, rabbit, cat, ferret, dog, cow, guinea pig, human	activates Na^+^/K^+^ channel	Saxena and Villalon, 1990; Page and McCubbin, 1955; Kaumann and Levy, 2006; Villalón and Centurión, 2007
		5-HT_2_	↓		G_q_	Parker et al., 1995; Pino et al., 1998; Linden et al., 1999; Centurion et al., 2002
		5-HR_4_	↑	Rat, pig, human		Wang et al., 2016
		direct modulation of SERCA				
Vasoactive intestinal peptide (VIP)	Neuropeptide co-released with ACh	VPAC1/2	↑	Rabbit, dog, guinea pig, human	G_s_	Frase et al., 1987; Hoover, 1989; Chang et al., 1994; Accili et al., 1996
Pituitary adenylate cyclase-activating polypeptide (PACAP)	Neurotransmitter and neuromodulator	PACAP 1-3 and VPAC1/2	↑/↓	Dog		Hirose et al., 1997a, b, 1998
			↑	Rat, human		Dorner et al., 1998; Whalen et al., 1999; Birk et al., 2007
Neuropeptide Y (NPY)	Neuropeptide co-released with NE		↑	Chick, rat, guinea pig		Lundberg et al., 1984; Beaulieu and Lambert, 1998; Jacques and Abdel-Samad, 2007
			↓	Dog, cat		Minkes et al., 1989; Chang et al., 1994
			none	Dog, rabbit		Allen et al., 1983; Rigel, 1988
Calcitonin gene-related peptide (CGRP)	Neuropeptide		↑	Rat, guinea pig, rabbit, dog, human^*iii*^	?	Struthers et al., 1986; Franco-Cereceda et al., 1987; Gennari et al., 1990; Bell and McDermott, 1996
Neurotensin	Neuromodulator	NTS1 and NTS2	↑	Rat, guinea pig, cat	G_s_ and G_q_	Chahl and Walker, 1981; Bachelard et al., 1985; Rioux et al., 1989
Somatostatin	Neuropeptide	SSTR 1-5	↓	Snake, human	all G_i_ and some G_i_ and G_q_	Donald et al., 1990; Bubinski et al., 1994
Natriuretic peptides: ANP	Autocrine, paracrine, and endocrine hormone	NPR-A and NPR-C	↑ or ↓ none	Dog, mouse, human, dog	GC (NPR-A) or Gi (NPR-C)	Biollaz et al., 1986; Bussien et al., 1986; Weidmann et al., 1986; Franco-Suarez et al., 1987; Nicholls and Richards, 1987 Volpe et al., 1987, 1990; Crozier et al., 1993
BNP		NPR-A and NPR-C	↑ none	Mouse mouse, dog	GC (NPR-A) or Gi (NPR-C)	Moghtadaei et al., 2016; Moghtadaei et al., 2016
CNP		NPR-B	↑	Dog, mouse	GC	Moghtadaei et al., 2016
Adenosine	Purine nucleoside	A1 and A3	↓	Human, dog, rabbit, rat, mouse, guinea pig	G_i_ and G_q_	West and Belardinelli, 1985; Belardinelli et al., 1988; Headrick et al., 2013; Lou et al., 2013, 2014; Li et al., 2017
		A2a and A2b	↑	Mouse	G_s_	Headrick et al., 2013
Angiotensin II (ANGII)	Hormone	AT_1_/AT_2_	↓	Guinea pig, rabbit	G_q_/11, G_s_, and G-protein independent pathways	Habuchi et al., 1995; Sheng et al., 2011
			↑	Dog		Lambert, 1995
			none	Human		Oney and Kaulhausen, 1982; Sander-Jensen et al., 1988
Endothelin (ET-1)	Hormone produced by endothelial cells	ET_A_ /ET_B_	↓/↑	Rabbit	G_i_ and *I*_KACh_ or G_q_ and NO	Tanaka et al., 1997; Ono et al., 2001
			↓	Guinea pig		Ishikawa et al., 1988
			↑	Cat		Minkes et al., 1989
			↓/↑	Pig		Gelzer et al., 2004
		ET_A_	↓?	Human^*i*^		Kolettis et al., 2003
		ET_B_	none?			
Thyroid hormones	Hormone produced by thyroid gland	Thyroid receptors	↑	Rat, mouse, human	activates transcription factors	Pachucki et al., 1999; Renaudon et al., 2000; Gloss et al., 2001; Roef et al., 2013; Rutigliano and Zucchi, 2017
Parathyroid hormones (PTH)	Hormone produced by parathyroid gland	PTH_1_R/PTH_2_R	↑	Rat, rabbit, human^*ii*^	G_s_ and G_q_	Hara et al., 1997; Shimoyama et al., 2001; Sugimoto et al., 2013
Bradykinin	Inflammatory peptide	BK2R	↑	Rabbit, rat, human^*iv*^	G_q_/11	Ponchon et al., 1995; Schaefer et al., 1996; Pian et al., 2007
			↓	Mouse		
Relaxin	Reproductive hormone	?	↑	Rabbit, rat	Increases cAMP and PKA	Kakouris et al., 1992; Han et al., 1994a
Nitric Oxide (NO)	Produced by NO synthase	Soluble guanylyl cyclase	↓	Human		Chowdhary et al., 2000
			↑	Human^*v*^		Chowdhary et al., 2002a

*^*i*^pre-existing CAD; ^*ii*^with heart failure; ^*iii*^healthy and heart failure; ^*iv*^with and without atherosclerosis; ^*v*^transplanted hearts.*

In this review, we first provide a brief overview of the mechanisms that drive SAN automaticity, to introduce the principal targets for modulation of SAN activity by neurohumoral signaling. This is followed by consideration of neurotransmitters and neuropeptides, and autocrine, paracrine, and endocrine factors with known effects on SAN activity and HR, including their receptors, intracellular signaling, and protein targets, in particular highlighting what is known from experimental models, what has been confirmed in human studies, and where gaps in our current understanding exist, to identify areas for future research.

## San Automaticity

Cardiac automaticity is driven by depolarization of SAN membrane potential during diastole (spontaneous diastolic depolarization, SDD; Figure 1). The principal mechanisms responsible for SDD have been heavily debated (Rosen et al., 2012). The two concepts that have emerged are that of a “membrane-clock,” resulting from a flow of ions through *trans*-sarcolemmal channels, and a “Ca^2+^-clock,” resulting from intracellular Ca^2+^ cycling (Bartos et al., 2015). It is now generally accepted that these are in fact overlapping and redundant systems that work together to cause SAN automaticity, and form a combined system often referred to as the “coupled-clock” (Lakatta et al., 2010; Rosen et al., 2012).

### Membrane-Clock

During early SDD, the membrane-clock is driven by an inward cation current known as the “funny” current (*I*_f_), passing through hyperpolarization-activated cyclic nucleotide-gated (HCN) channels (Difrancesco, 2010). There are four isoforms of HCN (HCN1-4), with HCN1, 2, and 4 expressed in the human heart, notably more prominently in the SAN than the atria, and with HCN1 specifically being expressed almost exclusively in the SAN (Li et al., 2015). Studies of HCN1 knock-out in mice support these findings, demonstrating high expression and critical importance of HCN1 for maintenance of HR and beat-to-beat stability (Fenske et al., 2013). Along with *I*_f_, inward Ca^2+^ currents through both Ca_v_3.1 transient (T-type, *I*_Ca_,_T_) and Ca_v_1.2/1.3 long-lasting (L-type, *I*_CaL_) channels are important for SAN firing (Mangoni and Nargeot, 2008). Ca_v_3.1 and Ca_v_1.3 activate at relatively low thresholds (∼ –70 and –55 mV, respectively), allowing them to contribute to the early portion of SDD (Mangoni and Nargeot, 2008). Once the threshold for Ca_v_1.2 activation is reached (∼ –40 mV), *I*_CaL_ generates the upstroke of the SAN AP [unlike working cardiomyocytes, in which the fast sodium (Na^+^) current (*I*_Na_), passing through Na_v_1.5 channels, is responsible for the AP upstroke] (Mesirca et al., 2015). Na_v_1.5 channels are heterogeneously expressed in the SAN, and primarily at the periphery, so while they are not necessary for SAN automaticity, they can indirectly influence pacemaker rate (Lei et al., 2005). Other heterogeneously expressed currents have been suggested to also contribute to SAN automaticity, such as the current passed by transient receptor potential-canonical (TRPC) channels (Ju et al., 2007) and a sustained inward current (*I*_st_) (Zhang et al., 2002), which has been postulated to represent the combined effects of *I*_CaL_ and current through the Na^+^-Ca^2+^ exchanger (*I*_NCX_) (Lakatta et al., 2010). In fact, the Na^+^-Ca^2+^ exchanger plays a fundamental role in SAN automaticity, and even though it could be included in the membrane clock as it generates a transmembrane current, it is fundamental in the “Ca^2+^-clock” system, so is discussed in the following section.

Another important consideration for SAN activity is the repolarizing currents, which determine the maximum diastolic membrane potential (MDP, the most negative occurring membrane potential), a key driver for *I*_f_. The rapid and slow delayed rectifier K^+^ currents (*I*_Kr_ and *I*_Ks_, respectively) are the primary repolarizing currents in SAN myocytes, with their decay at the end of the AP allowing for inward cation currents to cause SDD (Mangoni and Nargeot, 2008). Important for the occurrence of SDD, in primary SAN myocytes there is very little inward rectifier potassium (K^+^) current (*I*_K1_, passed by Kir2.1-2.4 channels) (Chandler et al., 2009), which in working myocytes stabilizes diastolic membrane potential at a resting value of ∼ –90 mV. It has also been suggested that the Ca^2+^-activated K^+^ channel current (*I*_K,Ca_) may be important for the early portion of SDD by maintaining MDP, and thus the driving force for *I*_f_, as their blockade can result in bradycardia or complete suppression of excitation (Weisbrod et al., 2016). There is evidence of other K^+^ currents in SAN myocytes that may contribute to repolarization, including: the transient outward K^+^ current (*I*_to_); the ultra-rapid delayed rectifier K^+^ current (*I*_Kur_); the acetylcholine (ACh)-activated K^+^ current (*I*_KACh_); and the adenosine triphosphate (ATP)-sensitive K^+^ current (*I*_K_,_ATP_) (Chandler et al., 2009). In addition, the inwardly rectifying chloride current (*I*_Cl_), which is activated late during the upstroke, as well as current through the Na^+^-K^+^ ATPase (which extrudes three Na^+^ ions while bringing two K^+^ ions into the cell, thus generating a net outward current) may contribute to SAN repolarization and the MDP (Mangoni and Nargeot, 2008).

### Ca^2+^-Clock

Intracellular Ca^2+^ cycling has been shown to be a fundamental contributor to SAN automaticity. During late SDD in SAN myocytes, there are localized spontaneous and/or Ca_v_1.3-triggered sub-sarcolemmal Ca^2+^ releases from the sarcoplasmic reticulum (SR) *via* ryanodine receptors (RyR), which are in close proximity to the Na^+^-Ca^2+^ exchanger (Lakatta et al., 2010; Torrente et al., 2016). These local Ca^2+^ releases are large in size and duration and rhythmic in nature (unlike Ca^2+^ sparks emerging from the SR in ventricular cells, which are smaller, stochastic events, Sirenko et al., 2013), and result in a depolarizing current as 1 Ca^2+^ ion is extruded from the cell in exchange for 3 Na^+^ ions through the Na^+^-Ca^2+^ exchanger (*I*_NCX_) (Lakatta et al., 2008). The importance of this mechanism for SAN firing has been demonstrated by *I*_NCX_ blockade in guinea pig and rabbit SAN myocytes (Bogdanov et al., 2001, 2006; Sanders et al., 2006), as well as by inhibition of RyR in guinea pig (Rigg et al., 2000), rabbit (Hata et al., 1996; Satoh, 1997; Bogdanov et al., 2001; Bucchi et al., 2007; Lyashkov et al., 2007), mouse (Masumiya et al., 2003; Ju et al., 2007; Nikmaram et al., 2008; Wu et al., 2009), and dog (Vinogradova et al., 2006; Joung et al., 2009). As a result, the rate and size of local Ca^2+^ releases, and the balance between Ca^2+^ reuptake into the SR [through the SR Ca^2+^-ATPase (SERCA) pump] and extrusion through the Na^+^-Ca^2+^ exchanger are key determinants of the rate of diastolic depolarization. Importantly, SAN myocytes have a higher basal level of cyclic adenosine monophosphate (cAMP), and thus protein kinase A (PKA)-dependent phosphorylation of Ca^2+^-handling proteins than working myocytes, resulting in a greater level of SR Ca^2+^ cycling, which facilitates Ca^2+^ clock function (Vinogradova et al., 2006). It has also been shown that SAN myocytes have higher basal levels of Ca^2+^/calmodulin dependent protein kinase II (CaMKII) compared to working myocytes, resulting in higher basal phosphorylation of L-type Ca^2+^ channels, RyR, and phospholamban (PLB), which when unphosphorylated inhibits SERCA (Vinogradova et al., 2000; Li et al., 2016).

### Coupled-Clock

The membrane- and Ca^2^-clocks together form a cohesive and robust system for SAN automaticity. Their coupling occurs *via* effects of one on the other and through the mutual entrainment of sarcolemmal ion channel activity and Ca^2+^ cycling by intracellular regulatory mechanisms (Lakatta et al., 2008). For instance, clock-coupling occurs as membrane repolarization by K^+^ channels (key to the membrane-clock) both activates *I*_f_ and affects intracellular Ca^2+^ balance (and in turn the Ca^2+^ clock) *via* voltage-dependent effects on *I*_CaL_ and *I*_N__C__X_ (Lakatta et al., 2010). *Vice versa*, *I*_NCX_ (essential for the Ca^2+^-clock) directly affects membrane potential (and thus voltage-dependent components of the membrane-clock) (Bogdanov et al., 2006). This also highlights that some currents, such as *I*_CaL_ and *I*_NCX_, are important components of both clocks, such that they are intricately linked. The fidelity of clock coupling is altered through simultaneous modifications of components within each system. For instance, neurohumoral alteration of PKA phosphorylation levels [through changes in adenylyl cyclase (AC) activity, the protein that converts ATP into cAMP] entrains the two clocks (Mattick et al., 2007; Vinogradova and Lakatta, 2009). In guinea pig and rabbit, this has been shown to be enhanced by Ca^2+^-activated isoforms of AC (important for basal AC activity in the absence of β-AR stimulation) found in SAN, but not working myocytes, which also acts to drive intracellular clock-coupling (Mattick et al., 2007; Younes et al., 2008).

And yet, one should note that oscillatory behavior, as occurs in the SAN, can result from a combination of activities that themselves are not oscillatory. In fact, neither *I*_f_, nor *I*_NCX_, nor the decay of *I*_K_ can in itself produce the oscillations in membrane potential that are key to the rhythmic excitation of the SAN. It is only their combined, out-of-phase activity that allows for SAN automaticity. Even ion fluxes not typically considered “pacemaker currents” are essential coupled-clock components, such as the flux generated by the Na^+^-K^+^ ATPase, which helps maintain electrochemical gradients and ionic homeostasis, which are crucial for SAN pacemaking (Sirenko et al., 2016). Thus, SAN automaticity may continue even with the loss of one (or more) of the currents described above, although important for the subject of this review, this may result in the loss of important neurohumoral control mechanisms.

### Species Differences in SAN Function

While the mechanisms of SAN automaticity described above are well conserved, there exist species-specific differences in SAN function potentially important for neurohumoral control of HR, which may provide some explanation for inconsistencies of experimental results. For instance, the range of basal HR across mammals is large, with an inverse relation to animal weight (Opthof, 2001). The normal resting HR of mouse is approximately 10-fold faster than human (∼700 *vs.* 70 beats/min), whereas rabbit falls somewhere between (∼250 beats/min). This is in part related to differences in the balance of sympathetic and parasympathetic nervous system control of the SAN. Although activation of both branches of the autonomic nervous system (ANS) alter HR in all species studied, there are species-differences in the relative contribution of the sympathetic and parasympathetic branches to basal HR. Specifically, in mouse and rat, resting HR is predominantly determined by sympathetic tone (Adachi et al., 2013), whereas in large animals, such as dog and human, HR is mostly determined by basal parasympathetic activity (Opthof, 2001). The predominance of sympathetic activity in mouse and rat is driven primarily by a need for non-shivering thermogenesis (*via* sympathetic stimulation of brown fat) when acclimated to room temperature, to maintain core body temperature (Axsom et al., 2019). At thermoneutral temperatures (∼30°C) sympathetic activity is reduced, unmasking parasympathetic input and significantly lowering HR (Axsom et al., 2019). Characteristics of the SAN AP also differ across species. In mouse, AP duration is ∼80 ms, in rabbit it is ∼200 ms, and in human it is ∼300 ms (Opthof, 2001). These differences in AP duration suggest relative differences in ionic current densities. It is known, for instance that while *I*_K1_ is almost entirely not present in rabbit SAN myocytes, it is active in some (but not all) mouse SAN pacemaker cells, although still with a much smaller current than in working cardiomyocytes (Cho et al., 2003). Further, differences in ionic current densities could relate to differences in post-translational modifications or activation and inactivation kinetics of various currents across species.

Important species-specific differences in intracellular Ca^2+^ handling also exist. In rabbit SAN myocytes it has been shown that Ca^2+^ release from the SR *via* RyR during SDD is spontaneous (Bogdanov et al., 2001), while in mouse Ca^2+^ release is instead trigged by Ca_v_1.3-mediated *I*_CaL_ (Torrente et al., 2016), and in cat by voltage-dependent activation of T-type Ca^2+^ channels (Hüser et al., 1996). In fact, α_1D_ (which is the pore forming subunit of C_av_1.3) L-type Ca^2+^ channels are present exclusively in the atria of mouse, rat, rabbit, and human and are thought to contribute to SDD, based on their activation at more negative membrane potentials than the most predominantly expressed L-type Ca^2+^ channel (with the α_1C_ pore-forming subunit), which is found throughout the heart (Mangoni et al., 2003; Qu et al., 2004). Removal of Ca^2+^ from the cytosol also differs, as in mouse and rat ∼92% of Ca^2+^ is pumped back into the SR by SERCA and only ∼7% is extruded through the Na^+^-Ca^2+^ exchanger, whereas in rabbit and human only 70% returns to the SR and 28% is extruded from the cell (Bers, 2000). As a result, *I*_NCX_ and its contribution to SDD is greater in rabbit/human than mouse/rat.

## Neurotransmitters and Neuropeptides

The SAN is strongly influenced by the ANS, with a variety of neurotransmitters [catecholamines such as norepinephrine (NE) and epinephrine, ACh, histamine, serotonin (5-HT)], as well as a host of neuropeptides (vasoactive intestinal polypeptide, calcitonin gene-related peptide, neuropeptide Y, neurotensin, and somatostatin) released by neurons in the heart that alter SAN activity and thus HR (Gordan et al., 2015).

In general, these substances bind to a GPCR, causing a conformational change in its cytoplasmic domains, which activates its subunits (G_α_ and G_βγ_) (Oldham and Hamm, 2008; Rosenbaum et al., 2009; Zhang et al., 2015). The G_α_ isoforms of primary importance in the SAN are G_αs_, G_αi/o_, and G_αq/11_ (Marin-Garcia, 2011). In the “stimulatory” G_αs_ pathway, activation of AC increases cAMP, which directly or indirectly (*via* PKA activation and subsequent phosphorylation) activates downstream targets (Gordan et al., 2015). When the “inhibitory” G_αi/o_ pathway is initiated, either G_αi_ or G_αo_ binds and inhibits AC, decreasing cAMP levels and preventing PKA activation (Gordan et al., 2015). Activation of G_αq/11_ on the other hand does not alter cAMP levels, but instead either G_αq_ or G_α11_ activates phospholipase C (PLC), which breaks down phosphatidylinositol 4,5-bisphosphate (PIP_2_) into diacylglycerol (DAG) and inositol triphosphate (IP_3_), both of which affect multiple downstream targets (Marin-Garcia, 2011). And while the G_α_ subunits initiate the primary signaling cascades, G_βγ_ subunits may also be involved (Zhang et al., 2015). Another important signaling pathway in SAN myocytes occurs through particulate or soluble guanylyl cylcase (GC) activation, which results in cGMP activation and modulation of downstream targets by protein kinase G (PKG) or other downstream regulatory molecules (Zaccolo and Movsesian, 2007).

### Catecholamines

Catecholamines (NE and epinephrine) are released from sympathetic neurons of the ANS (Gordan et al., 2015) (although both are also produced by the adrenal gland and can act as circulating hormones, or can be intrinsically generated by cells within the SAN) (Marin-Garcia, 2011; Moen et al., 2019). NE and epinephrine bind to α- and β-adrenergic receptors (AR). There are four subtypes of β-AR: β_1_-AR are the most abundant in the heart (75–80%); β_2_-AR are expressed in the heart, but to a much smaller extent than β_1_-AR, and are mostly compartmentalized in caveolae (Rybin et al., 2000); β_3_-AR are only minimally expressed in the heart; and β_4_-AR have not yet been thoroughly characterized (Madamanchi, 2007).

**FIGURE 2:**
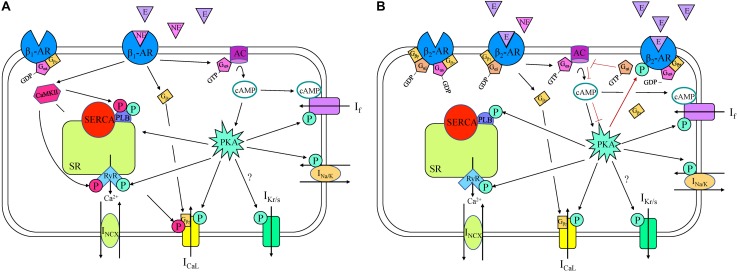
β_1_- **(A)** and β_2_- **(B)** adrenergic receptor (AR) signaling cascades in a sinoatrial node myocyte. AC, adenylyl cyclase; Ca^2+^, calcium; CaMKII, Ca^2^^+^/calmodulin-dependent protein kinase; cAMP, cyclic adenosine monophosphate; E, epinephrine; GDP, guanosine diphosphate; GTP, guanosine triphosphate; I_CaL_, long-lasting (L-type) Ca^2+^ current; I_f_, “funny” current; I_K__r__/__s_, rapid/slow delayed rectifier K^+^ current; I_Na/K_, Na^+^-K^+^ ATPase current; I_NCX_, Na^+^-Ca^2+^ exchanger current; NE, norepinephrine; P, phosphorylation; PKA, phosphokinase A; PLB, phospholamban; RyR, ryanodine receptor, SERCA, sarco/endoplasmic reticulum Ca^2^^+^-ATPase; SR, sarcoplasmic reticulum.

β_1_-AR are coupled to G_s_ proteins, so initiate the stimulatory AC/cAMP/PKA cascade (Figure 2), which has been shown to be essential for the increase in HR during adrenergic stimulation in rabbit SAN myocytes (Behar et al., 2016). β_1_-AR stimulation in the SAN increases HR through G_αs_-activation of AC, resulting in an increase in cAMP and PKA, which modulate the activity of various intracellular targets (Gordan et al., 2015). The combined action on these targets causes an increase in the rate of SDD, thus increasing SAN firing (Figure 1). For instance, in rabbit SAN myocytes the binding of cAMP to C-terminals of HCN channels causes a depolarizing shift in their activation kinetics, increasing open probability and *I*_f_ (DiFrancesco and Tortora, 1991; Barbuti and DiFrancesco, 2008). At the same time, PKA increases *I*_f_ by a phosphorylation-driven shift of its voltage-dependence of activation, as shown in mouse SAN myocytes (Liao et al., 2010). Although it is still not entirely clear whether cAMP and PKA work synergistically or independently on *I*_f_ during β_1_-AR stimulation, it is clear that they both cause an increase in HR (Larsson, 2010). Further, inhibition of *I*_f_ results in a moderate decrease in the effect of β-AR stimulation in rabbit (Noma et al., 1983; Vinogradova et al., 2002), mouse (Choate and Feldman, 2003), and rat (Yamamoto et al., 2006), demonstrating its key contribution to the chronotropic response. Yet, while it is apparent that an alteration in *I*_f_ during β_1_-AR stimulation modulates HR, it is still increased by β_1_-AR stimulation in animals in which HCN is deficient or absent, suggesting that other mechanisms are also involved (Vinogradova and Lakatta, 2009).

PKA also phosphorylates numerous other coupled-clock components contributing to SDD. Phosphorylation of the α_1_ subunit of L-type Ca^2+^ channels, purified from bovine hearts, increases their open probability, thus increasing current (De Jongh et al., 1996). It has also been shown that the G_αs_ subunit can directly stimulate L-type Ca^2+^ channels (Yatani et al., 1988) (T-type Ca^2+^ channels, on the other hand, are not affected by β_1_-AR stimulation in rabbit SAN myocytes, Hagiwara et al., 1988). Delayed-rectifier K^+^ channels show an increase in rate of deactivation and an increase in current amplitude following β_1_-AR stimulation in rabbit SAN myocytes, contributing to the increase in HR (Lei et al., 2000). β_1_-AR activation also increases Na^+^-K^+^ ATPase activity, which is an important compensatory mechanism for the maintenance of ionic homeostasis during increases in membrane currents (Shimoni, 1999) and it modulates *I*_Na_ and *I*_Cl_, however, the mechanism and the significance of this is uncertain, and likely neither of these contribute in a significant way to the β_1_-AR-related increase in HR (Mangoni and Nargeot, 2008). It has also been shown that there are phosphorylation sites on the Na^+^-Ca^2+^ exchanger, yet whether its phosphorylation occurs or is functionally relevant following β_1_-AR stimulation in the SAN remains unknown (Vinogradova and Lakatta, 2009).

Although some groups exclusively emphasize the role of effects on sarcolemmal currents in increased HR during β_1_-AR activation (Bucchi et al., 2007; Himeno et al., 2008), there is also evidence demonstrating a role of alterations in Ca^2+^-clock components in the response (Lyashkov et al., 2018). Specifically, the effect of RyR inhibition by ryanodine on the chronotropic response following β_1_-AR stimulation has been extensively studied in a multitude of species. In rabbit SAN cells, β_1_-AR activation increases the frequency and amplitude of local Ca^2+^ releases during SDD, which increases *I*_NCX_, the rate of SDD, and in turn, AP frequency. This effect is eliminated when RyR are inhibited by ryanodine, despite a preserved increase in L-type Ca^2+^ current amplitude (Vinogradova et al., 2002). Although this result suggests that the target of β_1_-AR activation is indeed the Ca^2+^-clock, another study in rabbit SAN has suggested that the alteration of Ca^2+^ homeostasis by RyR suppression has an indirect effect on HR, by disrupting the increase in cAMP near HCN channels (Bucchi et al., 2003). Either way, studies in the mouse (Wu et al., 2009), guinea pig (Rigg et al., 2000), and dog (Vinogradova et al., 2006; Joung et al., 2009) have all demonstrated that suppression of RyR with ryanodine diminishes the chronotropic response to β_1_-AR stimulation, indicating a critical role for Ca^2+^ release from the SR. β_1_-AR activation also increases PLB phosphorylation in SAN cells (Vinogradova et al., 2006), releasing its inhibition on SERCA and causing more rapid Ca^2+^ re-uptake into the SR (Marin-Garcia, 2011), which increases SR Ca^2+^ content.

Basal levels of CaMKII-dependent phosphorylation of RyR, PLB, and L-type Ca^2+^ channels are present in rabbit (but not mouse) SAN myocytes (Vinogradova et al., 2000; Wu and Anderson, 2014; Li et al., 2016), suggesting a species-dependence of the role that CaMKII plays in the maintenance of basal HR. In both mouse and rabbit, however, CAMKII is important under physiological stress, with CAMKII levels increased by sympathetic stimulation (Wu and Anderson, 2014). For instance, when CaMKII is inhibited, mice are less responsive to β_1_-AR stimulation (Wu et al., 2009).

While sympathetic stimulation in the SAN primarily acts through β_1_-AR, automaticity may also be affected by activation of β_2_-, β_3_-, and α_1_-AR (Marin-Garcia, 2011). β_2_-AR are coupled to both G_αs_ and G_αi_ subunits, activating the G_αs_ cascade under normal physiological conditions, but under other conditions can switch to the G_αi_ cascade (Paur et al., 2012; Machuki et al., 2018). For example, when high levels of epinephrine are released from the adrenal gland during stress, binding to β_2_-AR initially activates the G_αs_ subunit (as demonstrated in ventricular myocytes), triggering the same signaling pathways as β_1_-AR stimulation, thus increasing HR (Figure 2). The resulting PKA produced by this signaling cascade, however, phosphorylates the receptor, initiating a switch from G_αs_ to G_αi_ activation (Fischmeister et al., 2006). Once the G_αi_ subunit is activated, AC activity is inhibited, decreasing intracellular cAMP, PKA, and HR (Madamanchi, 2007). In the SAN, these actions are facilitated by the co-localization of β_2_-AR with HCN4 channels in caveolae, thus restricting the increase in cAMP to specifically modulate *I*_f_ [the close proximity of the receptor and the effector allows for this compartmentalization by phosphodiesterases (PDEs)] (Barbuti and DiFrancesco, 2008). In contrast, in the piglet and rat SAN it has been shown that β_2_-AR are important in mediating the response to NE and epinephrine independent of PDE compartmentalization (Christ et al., 2009; Galindo-Tovar et al., 2010).

β_3_-AR have also not been thoroughly studied in the SAN and therefore the role they play in HR modulation is uncertain. One complicating factor is that different isoforms of β_3_-AR have been shown to differentially couple to G_αs_ or G_αi_, although the existence of β_3_-AR isoforms have not been characterized in the heart (Machuki et al., 2018). Furthermore, likely due to the variable G-protein binding abilities of β_3_-AR, responses to β_3_-AR agonists are variable throughout the heart (Dessy and Balligand, 2010).

Catecholamines also bind to α-AR in the heart. α-AR are coupled to G_q/11_-proteins, which activate PLC (Jensen et al., 2014), causing hydrolysis of PIP_2_ into IP_3_ and DAG (Marin-Garcia, 2011). When IP_3_ binds to IP_3_R on the SR membrane, this causes release of Ca^2+^, increasing cytosolic Ca^2+^ levels, which causes the further release of Ca^2+^ from the SR through RyR (Marin-Garcia, 2011). Three IP_3_R isoforms have been identified in the mouse SAN, and it has been shown that their activation can modulate automaticity *via* Ca^2+^-dependent mechanisms (Ju et al., 2011, 2012; Kapoor et al., 2015). DAG, on the other hand activates PKC, which phosphorylates various targets such as L-type Ca^2+^ channels and the Na^+^-Ca^2+^ exchanger (Baker, 2014). Yet some studies of the effects of α-AR in the SAN are conflicting. For instance, in isolated SAN from both young and adult rabbits, phenylephrine (which activates α-AR) does not alter HR (Hewett and Rosen, 1985). Despite this result, greater expression of α-AR occurs in the SAN compared to surrounding myocardium in rat, suggesting a role for α-AR in HR control (Saito et al., 1994).

The control of HR by the sympathetic ANS is well documented in humans, as stimulation of sympathetic neurons in the human SAN increase HR and blockade of sympathetic receptors decreases HR (James et al., 1970), but many of the mechanistic details observed in experimental model systems have yet to be confirmed. β_1_- and β_2_-AR are both predominant in the human SAN, and β_2_-AR expression is more than two times higher in the SAN than the surrounding atrial tissue (Rodefeld et al., 1996). In the isolated human SAN isoproterenol has been shown to decrease cycle length and shorten AP duration by increasing the rate of repolarization, while also hyperpolarizing the MDP, consistent with what has been shown in animal models (Drouin, 1997). Yet, in contrast to what has been shown in animals, administration of phenylephrine in human causes either an increase (a response that is attenuated with age) (Saitoh et al., 1995) or a decrease in HR (Mary-Rabine et al., 1978; Drugge et al., 1985).

### Acetylcholine

**FIGURE 3:**
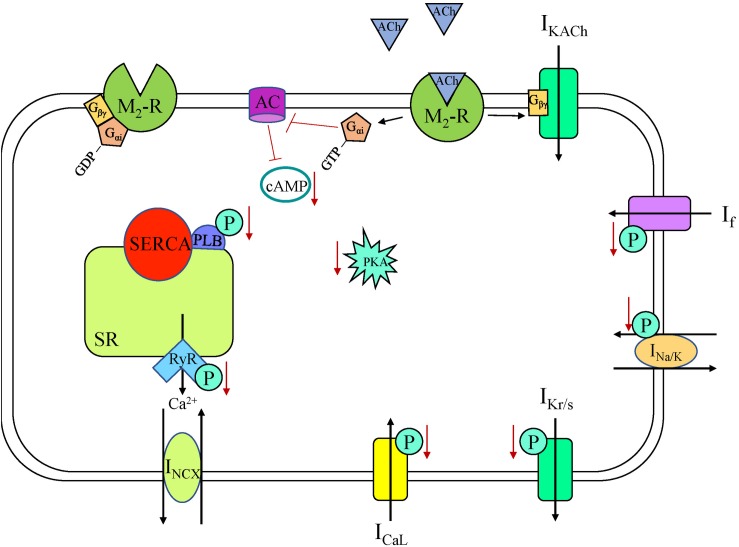
Muscarinic acetylcholine receptor (M_2_-R) signaling cascade in a sinoatrial node myocyte. AC, adenylyl cyclase; ACh, acetylcholine; cAMP, cyclic adenosine monophosphate; I_CaL_, long-lasting (L-type) Ca^2+^ current; I_f_, “funny” current; I_KACh_, acetylcholine-activated K^+^ current; I_Kr/s_, rapid/slow delayed rectifier K^+^ current; I_Na/K_, Na^+^-K^+^ ATPase current; I_NCX_, Na^+^-Ca^2+^ exchanger current; P, phosphorylation; PKA, phosphokinase A; PLB, phospholamban; RyR, ryanodine receptor, SERCA, sarco/endoplasmic reticulum Ca^2^^+^-ATPase; SR, sarcoplasmic reticulum.

The parasympathetic branch of the ANS counterbalances sympathetic effects. Parasympathetic neurons release ACh, which binds to muscarinic receptors in the sarcolemma (Gordan et al., 2015). There are five muscarinic subtypes that have been identified, but it is the M_2_-receptor (M_2_R) that elicits the functional response in the human heart (Harvey, 2012). In the SAN, ACh activation of M_2_R (a GPCR) leads to decreased HR as it is coupled to G_i_-proteins (Figure 3; Harvey, 2012). In rabbit SAN myocytes, this decrease in HR is associated with a negative shift of MDP, a decrease in SDD rate, and an increase in AP duration (Verkerk et al., 2012; Figure 1). Multiple M_2_R-mediated pathways have been shown in rabbit SAN cells to produce these effects over different time scales and with different ACh concentrations: activation of *I*_KACh_, inhibition of *I*_f_, and inhibition of Ca^2+^-clock components (Accili et al., 1998). *I*_KACh_ is the fastest-muscarinic pathway, with the response occurring in less than 2 s in rabbit SAN cells, as no cytosolic components are required for current activation (Accili et al., 1998). Specifically, again in rabbit SAN cells, when ACh binds M_2_R the G_βγ_ subunit activates G-protein regulated K^+^ (GIRK) channels, with the ensuing efflux of K^+^ causing a negative shift of MDP, slowing HR (Renaudon et al., 1997). GIRK1 and GIRK4 are both expressed in the heart and work together to form the channel (two α-subunits of each isoform) (Marin-Garcia, 2011), although in the human SAN GIRK expression is heterogeneous, with GIRK4 expression being highest in the center of the SAN (Li et al., 2017). Interestingly, SAN myocytes from GIRK4 knockout (KO) mice have a similar basal HR compared to control, but GIRK4 is indispensable for the generation of *I*_KACh_, which accounts for approximately half of the HR reduction provoked by vagal stimulation (Wickman et al., 1998).

While direct binding of the G_βγ_ subunit to GIRK elicits a response, multiple mechanisms for how this activates the channel have been proposed, based on data from *Xenopus* oocytes. One suggestion is that GIRK channels are intrinsically inhibited by a terminal domain of GIRK1 protein and binding of G_βγ_ removes inhibition and opens the channel (Reuveny et al., 1994). Another is that GIRK is activated by PIP_2_, but with a low sensitivity, and that G_βγ_ binding increases the affinity of GIRK for PIP_2_, thus increasing current activation (Huang et al., 1998). It is unclear, however, why the G_βγ_ subunit of G_s_ proteins do not activate *I*_KACh_ (Marin-Garcia, 2011). This lack of response may be due to compartmentalization of M_2_R and GIRK channels, but this possibility requires further investigation.

The slower components causing decreased HR upon M_2_R activation are second messenger cascades. The activated G_αi_ subunit reduces AC activity in rabbit SAN cells, resulting in decreased cAMP levels, fewer active HCN channels (due to a negative shift in the voltage dependence of activation of I_f_), and a reduction in the SDD slope (Accili et al., 1998; Van Borren et al., 2010). Activation of M_2_R with carbachol in rabbit SAN myocytes has been shown to have a dose-dependent effect on PLB phosphorylation and Ca^2+^-cycling via a G_i_-dependent pathway (Lyashkov et al., 2009). The negative chronotropic effect of ACh was shown to be dependent upon cAMP/PKA-dependent phosphorylation both in single rabbit SAN cells and in a computational rabbit SAN cell model, through effects on multiple components of the coupled-clock system (Yaniv et al., 2013; Behar et al., 2016). At low concentrations carbachol has been shown to decrease HR without activating *I*_KACh_, acting only *via* a cAMP-mediated, PKA-dependent suppression of Ca^2+^ signaling (Lyashkov et al., 2009). With higher concentrations, carbachol additionally decreases HR *via* activation of *I*_KACh_ (Lyashkov et al., 2009). These data demonstrate the importance of Ca^2+^ -clock suppression in the reduction in HR. Activation of M_2_R has also been shown to be coupled with NO synthesis, mediated by endothelial NO synthase (eNOS) (Han et al., 1994b). NO generation activates GC receptors, causing an increase in cGMP levels, PDE2 activation, inhibition of cAMP, reduced L-type Ca^2+^ channel phosphorylation, and ultimately decreased *I*_CaL_ (Han et al., 1995). Overall, the combination of the direct effects of M_2_R stimulation, including a more negative MDP due to *I*_KACh_ activation and a slower SDD due to reduced *I*_f_ and Ca^2+^ cycling, along with indirect NO/cGMP-mediated effects, leads to a decrease in HR in response to ACh release.

Although a vast amount of research has been done regarding the intracellular mechanisms for parasympathetic control of HR in the SAN of many experimental animals, the translation to humans is somewhat lacking. It is known that M_2_R density is nearly three times higher in the human SAN compared to surrounding atrial myocardium (Rodefeld et al., 1996) and that GIRK protein expression follows a similar trend (Li et al., 2017). In human isolated SAN, ACh increases cycle length and negatively shifts the MDP (Drouin, 1997). Direct stimulation of parasympathetic ganglia innervating the human SAN similarly causes a cycle length prolongation (Quan et al., 1999), and anticholinergic drugs slow HR (Nwazue et al., 2014). Although the HR effect of parasympathetic ANS activity is well characterized in human, some of the specific mechanisms demonstrated in animal models remain unconfirmed due to limitations of experimentation. This has been supplemented by the use of human-specific SAN cell computational models, which suggest that the voltage-dependence of M_2_R has significant effects on SDD, and that changes in the voltage-sensitivity of M_2_R may provide a mechanistic explanation for the increased sensitivity to changes in vagal tone in certain disease states (Moss et al., 2018).

### Histamine

Histamine released from neurons acts as a neurotransmitter but can also act as a hormone when released from mast cells and basophils in the heart (Marin-Garcia, 2011). There are four types of histamine receptors, all of which are GPCRs, with H_1_R coupled to the G_q/11_ pathway activating PLC, H_2_R coupled to the G_s_ cascade activating cAMP and PKA, and both H_3_R and H_4_R coupled to G_i_ signaling (Hattori et al., 2004). Histamine receptor expression is highly variable across species and cardiac tissues, and although both H_1_R and H_2_R are expressed in the atria of rat, guinea pig, rabbit, and human hearts, H_2_R is most abundant and dominates cardiac responses (Matsuda et al., 2004), including in the SAN, where H_2_R stimulation increases HR *via* increased cAMP and PKA levels (Kevelaitis et al., 1994; Hattori et al., 2004). In rabbit SAN tissue this increase in HR is associated with a shift in the primary pacemaker site (Kevelaitis et al., 1994). In rabbit isolated SAN myocytes, histamine increases firing *via* H_2_R activation, and at high concentrations induces arrhythmias (Satoh, 1993). Interestingly, resident cardiac mast cells, the main supply of histamine in the heart, predominantly reside in close proximity to the SAN (Nistri et al., 2008). When these mast cells become activated due to an inflammatory response, such as during acute myocardial infarction or septic shock, they release histamine, which has been shown to cause severe tachyarrhythmias in pigs (Matsuda et al., 2004; Nistri et al., 2008). Yet, the role of histamine in the heart is complex, as activation of H_3_R can inhibit the arrhythmogenic increase in NE release from sympathetic nerve terminals that is associated with ischemia (Levi and Smith, 2000). Not only does histamine alter HR during an inflammatory response, it also has been shown to increase HR in the initial stages of digestion in pythons, a useful model system for postprandial tachycardia due to the magnitude and infrequency of their meals (Skovgaard et al., 2009). Although these studies and the varying receptor subtypes, signaling cascades, and receptor locations (both across tissue types and in nerve terminals) demonstrate the potential for a subtle and more complex role for histamine in HR control, the positive chronotropic effect *via* H_2_R stimulation is the most commonly reported finding, particularly in human (Wolff and Levi, 1986; Hu et al., 1997; Hattori et al., 2004; Matsuda et al., 2004). In fact, it has been suggested that that administration of H_2_R inhibitors could be used to decrease HR following autonomic blockade in people with SAN dysfunction (Hu et al., 1997).

### Serotonin

The chronotropic response to serotonin (5-HT) is complex, as it binds to many different receptor types, both directly on cardiac tissue and also on autonomic nerve terminals, causing bradycardia, tachycardia, or sometimes both (Linden et al., 1999). There are at least fourteen different receptor types expressed in the heart which elicit inconsistent responses (Saxena and Villalon, 1990).

Many experimental studies have shown 5-HT to cause a decrease in HR. Intravenously administered 5-HT in the rat, rabbit, cat, ferret, dog, cow, and guinea pig causes an acute decrease in HR that can be blocked by vagotomy or atropine (James, 1964; Kaumann and Levy, 2006; Villalón and Centurión, 2007). This response is largely due to activation of 5-HT_3_-type receptors on parasympathetic neurons, which are coupled to a ligand-gated Na^+^/K^+^ channel. When opened, this channel causes depolarization, neurotransmitter release, and reflex bradycardia (Saxena and Villalon, 1990). Bradycardia can also be the result of 5-HT binding to 5-HT_1_ receptors on sympathetic neurons, causing their inhibition, or on parasympathetic neurons, causing their stimulation (Villalón and Centurión, 2007).

Conversely, an increase in HR with 5-HT has also been demonstrated experimentally. For example, in pithed rats, it has been shown that circulating 5-HT increases HR by binding to 5-HT_2_ receptors in the myocardium or on presynaptic sympathetic neurons (Gothert et al., 1986; Centurion et al., 2002). The 5-HT_2_ receptor is a G_q_-protein coupled receptor, and activates the PLC signaling cascade (Villalón and Centurión, 2007). It was also recently shown in rat cardiomyocytes combined with computational modeling that SERCA can be serotonylated (receptor-independent activation directly by 5-HT), which alters SERCA’s affinity for Ca^2+^ and represents a potential mechanism for direct effects of 5-HT on SAN automaticity that warrants further investigation (Wang et al., 2016). It appears that intravenously administered 5-HT in pig, on the other hand, causes tachycardia *via* activation of 5-HT_4_ receptors, as inhibition of 5-HT_4_ abolished this response (Parker et al., 1995).

In humans, intravenously administered 5-HT has been shown to both increase or decrease HR, consistent with the variable response seen in animal models (Page and McCubbin, 1955; Kaumann and Levy, 2006). Although it has not been demonstrated experimentally, it is likely that bradycardia is the result of 5-HT_3_ receptor activation on parasympathetic neurons as demonstrated in animal models. Contrarily, the tachycardia is hypothesized to be due to activation of 5-HT_4_ receptors, as a number of studies have identified 5-HT_4_ antagonists that inhibit the 5-HT induced increase in contractility seen in the human right atrium (Kaumann et al., 1990, 1991, 1994; Kaumann, 1993; Parker et al., 1995). 5-HT_4_ receptor activation increases contractility *via* an increase in cAMP, and it is feasible that 5-HT would also increase HR in the SAN through a 5-HT_4_ receptor activation mediated increase in cAMP (Kaumann et al., 1991). This is further supported by the observation that 5-HT_4_ receptor activation increases *I*_f_ in human atrial myocytes, which might be expected to also occur in SAN myocytes (Pino et al., 1998). Interestingly, in humans it has also been shown that chronic treatment with β-blockers increases sensitivity to 5-HT in the atria, implicating cross-talk between 5-HT_4_ receptors, β-AR, and M_2_R (Sanders et al., 1995). 5-HT has also been implicated in a vast array of human pathological cardiac conditions, which is unsurprising given the large number of different receptor types and their locations (Kaumann and Levy, 2006). Overall, while many studies have assessed the effect of 5-HT on HR, a consistent effect on HR in humans and the mechanisms eliciting these responses remain to be clearly elucidated.

### Vasoactive Intestinal Polypeptide

Vasoactive intestinal polypeptide (VIP) is a neuropeptide co-released with ACh from parasympathetic neurons, yet has the opposite effect of ACh, increasing HR (Mangoni and Nargeot, 2008). VIP binds to two receptor subtypes, VPAC1 and VPAC2, both of which are GPCRs that activate G_s_-protein cascades, stimulating AC to produce cAMP, which triggers PKA (Marin-Garcia, 2011). VIP application on rabbit SAN myocytes and canine Purkinje fibers positively shifts the activation curve of *I*_f_, which increases the rate of SDD and SAN firing (Chang et al., 1994; Accili et al., 1996). A dose-dependent increase in HR has been observed in guinea pig isolated hearts following bolus injections of VIP (Hoover, 1989). Similarly, when VIP is given intravenously to healthy humans, there is a dose-dependent increase in HR (Frase et al., 1987). Yet, still little is known about the role of VIP in human HR regulation (Henning and Sawmiller, 2001). Because VIP is released along with ACh but removed more slowly from the synapse, it is thought to be important for moderating the ACh-induced decrease in HR, contributing to the tachycardia seen after vagal stimulation (Mangoni and Nargeot, 2008), and possibly protecting against neutrally mediated bradycardia (Henning and Sawmiller, 2001). Interestingly, the affinity, density, responsiveness of VIP receptors changes in heart failure and hypertension, warranting further investigation of its role in disease progression and prevention in humans (Henning and Sawmiller, 2001).

### Pituitary Adenylate Cyclase-Activating Polypeptide

Pituitary adenylate cyclase-activating polypeptide (PACAP) is a neurotransmitter and modulator which is very closely related to VIP (Henning and Sawmiller, 2001). It binds to three GPCR subtypes, two of which are identical to VPAC1 and VPAC2 (Hirose et al., 1997a). In a group of studies done in the dog (under anesthesia, in the isolated heart, and in the atria), PACAP caused a biphasic chronotropic response, with an initial increase, followed by a decrease in HR (Hirose et al., 1997a, b, 1998). In the anesthetized dog, the PACAP-induced decrease in HR is abolished following M_2_R blockade, indicating that the change in HR is likely due to activation of parasympathetic nerves (Hirose et al., 1997a). Interestingly, in the dog it appears that the increase in HR with PACAP is not mediated through β-ARs (as the response is not attenuated by β-AR blockade), but is due to activation of PACAP receptors directly on the tissue (Hirose et al., 1997a). The specific intracellular pathways eliciting this response remain unknown, however, in other tissues PACAP has been reported to increase cAMP, which could explain the positive chronotropic response. Conversely, in the anesthetized rat, HR increases with PACAP administration and the response is abolished by β-AR blockade, indicating that in this species the positive chronotropic response is mediated by neuromodulation of sympathetic neurons, rather than a direct effect on SAN tissue (Whalen et al., 1999). Although not much is known about the role of PACAP in the human SAN, in two separate studies on young, healthy humans, PACAP caused an increase in HR (Dorner et al., 1998; Birk et al., 2007).

### Neuropeptide Y

Neuropeptide Y (NPY) is co-localized with NE and expressed by sympathetic neurons innervating the cardiovascular system (Marin-Garcia, 2011). NPY binds to a group of GPCRs (Y_1_, Y_2_, and Y_5_) which activate the G_i/o_ signaling cascade (Marin-Garcia, 2011). Chronotropic responses vary across species and experimental preparations, with NPY having been shown to cause inconsistent changes in HR (Beaulieu and Lambert, 1998). NPY pre-synaptically inhibits NE release from sympathetic neurons, but can also potentiate post-synaptic effects of NE, therefore working both with and against sympathetic nervous system stimulation of the SAN (Beaulieu and Lambert, 1998). In embryonic chick ventricular myocytes, NPY increases spontaneous firing frequency by increasing stimulation of L-type Ca^2+^ channels *via* activation of Y_1_ receptors (Jacques and Abdel-Samad, 2007). An increase in firing rate is observed with NPY application in guinea pig isolated atria and rat isolated hearts (Lundberg et al., 1984; Beaulieu and Lambert, 1998). Conversely, in canine Purkinje fibers, NPY reduces *I*_f_ (either *via* a decrease in maximal *I*_f_ conductance or *via I*_f_ current blockade, rather than altering the voltage-dependence) causing decreased HR (Chang et al., 1994). Similarly, in anesthetized cats, NPY injection causes a decrease in HR (Minkes et al., 1989). In Langendorff perfused rabbit hearts and in anesthetized dogs no change in HR is observed with NPY application (Allen et al., 1983; Rigel, 1988). Differences in experimental models might explain some of these differences, however, the specific actions of NPY on SAN myocytes requires further investigation. Very little is known about the impact of NPY on the human SAN. A correlation between endogenous NPY and tachycardia was found in adults with heart failure, however, whether this correlation is causative or the change in HR is a confounding variable is unknown (Hulting et al., 1990). Contrarily, in children with vasovagal syncope no correlation was found between NPY levels and HR (Liao et al., 2017). NPY has been investigated broadly for its role in many cardiovascular diseases, but regarding its effect on HR in human, further investigation is needed.

### Calcitonin Gene-Related Peptide

Calcitonin gene-related peptide (CGRP) has been shown to increase HR in many different expression systems, however, whether it is acting directly on the SAN to alter HR and/or as a neuromodulator with indirect chronotropic effects is unclear (Beaulieu and Lambert, 1998). Tachycardia has been observed in response to intravenous administration of CGRP in many species including the rat, rabbit, dog, and in humans (Bell and McDermott, 1996). In neonatal rat isolated cardiomyocytes and rat and guinea pig isolated right atria CGRP increases beating rate, and in rats intravenous CGRP increases HR even in the presence of autonomic antagonists, suggesting a direct action of CGRP on the SAN (Marshall et al., 1986; Fisher et al., 1988). Conversely, despite HR being increased following intravenous administration of CGRP in the rabbit, in rabbit isolated right atria no change in beating rate is observed, suggesting an effect on ANS ganglia rather than directly on SAN myocytes (Holman et al., 1986). In the dog, CGRP also seems to act as a neuromodulator, as when given intravenously it does not affect HR at baseline, but does attenuate the reduction of HR with vagal stimulation, a response that is abolished by sympathectomy and parasympathectomy (Rigel et al., 1989). In humans, a higher amount of CGRP is found in the atria than ventricles, and in the SAN than the rest of the atria, with CGRP binding sites found throughout the heart (Beaulieu and Lambert, 1998). In three different clinical studies, two involving healthy individuals and one with patients in heart failure, intravenously administered CGRP increased HR, even in the presence of adrenergic receptor blockade (Struthers et al., 1986; Franco-Cereceda et al., 1987; Gennari et al., 1990). However, whether the CGRP-mediated increase in HR in humans is exclusively due to the direct action on the SAN or if CGRP also modulates HR through neuromodulation of ANS ganglia requires further investigation.

### Neurotensin

Neurotensin is a neuromodulator with many targets within the brain, but also the heart, including the SAN (Marin-Garcia, 2011). Neurotensin receptor subtypes NTS_1_ and NTS_2_ are expressed in the heart and both are GPCRs (Osadchii, 2015). NTS_1_ has been shown to activate PLC and PKA through the G_q/11_ and G_s_ signaling pathways, respectively (Marin-Garcia, 2011). It has been hypothesized that neurotensin modulates cardiovascular responses primarily *via* NTS_1_ (Osadchii, 2015)_._ Neurotensin has been shown to increase HR in anesthetized rat, guinea pig, and cat, and in guinea pig isolated hearts and atria (Osadchii, 2015). The increase in HR in both the anesthetized guinea pig and the guinea pig isolated heart was not abolished even in the presence of a multitude of autonomic antagonists, suggesting that neurotensin can act directly on the SAN, not simply by altering neuronal activation (Bachelard et al., 1985; Rioux et al., 1989). Conversely, in the anesthetized rat the response was abolished by adrenergic blockade, suggesting the response may be neurally mediated (Chahl and Walker, 1981). Although it’s feasible to predict that neurotensin would also increase HR in humans, through either direct action on the SAN or on ANS ganglia within the atria, currently no data exists. As levels of endogenous neurotensin increase in some pathological conditions it is of potential importance to establish the impact of neurotensin on the human SAN.

### Somatostatin

Similar to the other neuropeptides described above, somatostatin may act both directly on cardiovascular tissue through its own receptors, while also modulating the release of catecholamines or ACh from autonomic neurons (Marin-Garcia, 2011). Somatostatin activates five GPCRs, all of which stimulate the G_i/o_ pathway, and some of which also activate G_q/11_ cascades (Marin-Garcia, 2011). In snake isolated atrial preparations somatostatin causes a decrease in HR (Donald et al., 1990). Similarly, in a clinical study involving patients admitted for unexplained heart palpitations, intravenous somatostatin decreased HR (Bubinski et al., 1994). It is unclear if this effect is due to the direct action of somatostatin on SAN myocytes or through modulation of autonomic inputs to the SAN. No other data regarding the chronotropic effect of somatostatin on HR exists, and therefore this is an area requiring further investigation.

## Autocrine, Paracrine, and Endocrine Factors

Various peptides, such as the neuropeptides described above, are important modulators of HR. Many are produced by cells within the heart, acting as autocrine and paracrine factors, while others are produced elsewhere (e.g., in the brain, kidneys, liver, or vasculature) and reach the heart through the circulation, thus acting as endocrine factors (Beaulieu and Lambert, 1998). Their importance for HR regulation is an active area of investigation, and crucial for a complete understanding of SAN control by neurohumoral agents.

### Natriuretic Peptides

**FIGURE 4:**
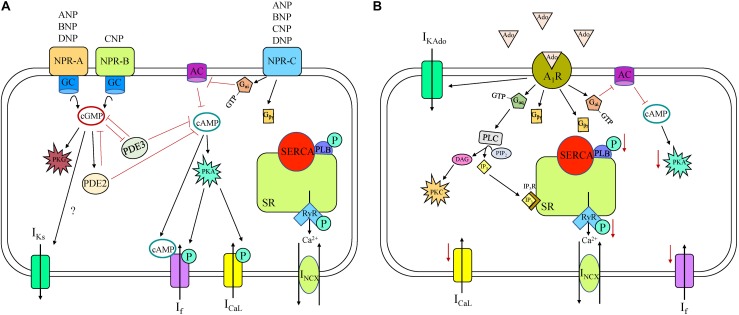
Natriuretic peptide receptor (NPR) **(A)** and adenosine **(B)** receptor signaling cascade in a sinoatrial node myocyte. A_1_R, adenosine receptor 1; AC, adenylyl cyclase; ANP, atrial natriuretic peptide; BNP, brain natriuretic peptide; cAMP, cyclic adenosine monophosphate; cGMP, cyclic guanosine monophosphate; CNP, C-type natriuretic peptide; DAG, diacylglycerol; DNP, *Dendroaspis* natriuretic peptide; GC, guanylyl cyclase; I_CaL_, long-lasting (L-type) Ca^2+^ current; I_f_, “funny” current; I_KAdo_, adenosine-induced inwardly rectifying K+ current; I_Ks_, slow delayed rectifier K^+^ current; I_NCX_, Na^+^-Ca^2+^ exchanger current; IP_3_, inositol triphosphate; IP_3_R, inositol triphosphate receptor; P, phosphorylation; PDE, phosphodiesterase; PIP_2_, Phosphatidylinositol 4,5-bisphosphate; PKA, phosphokinase A; PKC, phosphokinase C; PKG, phosphokinase G; PLB, phospholamban; PLC, phospholipase C; RyR, ryanodine receptor, SERCA, sarco/endoplasmic reticulum Ca^2^^+^-ATPase; SR, sarcoplasmic reticulum.

Natriuretic peptides (NP) are a family of peptide hormones that are important in cardiovascular physiology and disease. There are four NP family members: atrial NP (ANP); brain NP (BNP); C-type NP (CNP), and *Dendroaspis* NP (DNP) (Moghtadaei et al., 2016). NP can be produced and stored in atrial myocytes and released during atrial stretch or produced and released from cardiac fibroblasts. All four NPs are present in the circulation, but NP concentrations are much higher in the myocardium than the circulation, due to their local production and perform autocrine and paracrine functions. NP bind to three NP receptors (NPR): NPR-A; NPR-B; and NPR-C, all of which are expressed in the SAN. ANP, BNP, and DNP all bind to NPR-A, whereas only CNP binds to NPR-B. Both NPR-A and NPR-B are particulate (membrane bound) GC receptors, meaning ligand binding to the extracellular NPR activates intracellular GC enzymes and increases intracellular cGMP concentrations (Figure 4). cGMP activates protein kinase G (PKG) and PDE2 and inhibits PDE3. PKG can phosphorylate many components of SAN automaticity, while PDEs hydrolyze cAMP and cGMP and are therefore important modulators of cyclic nucleotide activity. Unlike the other NPR subtypes, NPR-C is not a GC receptor but an atypical GPCR with a single transmembrane domain (unlike a classical GPCR with seven transmembrane domains) that activates a specific G_i_-protein signaling cascade that inhibits AC, thus decreasing cAMP levels.

Numerous studies have been performed using various animal models to assess the chronotropic effects of NP. ANP has been shown to decrease HR in rat and not effect HR in dog, BNP has been shown to increase HR in mouse and not effect HR in mouse and dog, and CNP has been shown to increase HR in dog and mouse (Moghtadaei et al., 2016). BNP and CNP increase HR by increasing *I*_f_ and *I*_CaL_ and shifting the voltage dependence of activation of both currents (Springer et al., 2012). Interestingly, the effects of BNP and CNP on *I*_f_ and *I*_CaL_ are indistinguishable between NPR-C KO and wild-type mice, revealing that activation of NPR-C does not modulate automaticity under basal conditions. Conversely, blocking NPR-A or inhibiting PDE3 both abolish the effects of BNP or CNP on *I*_f_ and *I*_CaL_ (Springer et al., 2012). These data indicate that under basal conditions, BNP and CNP increase HR by way of NPR-A and NPR-B (not NPR-C), by activating GC, increasing cytosolic cGMP, and inhibiting PDE3, all of which lead to higher cAMP concentrations. In guinea pig SAN myocytes, application of ANP causes an increase in *I*_Ks_ through an increase in cGMP (Shimizu et al., 2002). Together, these results indicate that under basal conditions, NPR-A and NPR-B activation have a greater influence on SAN automaticity than NPR-C.

Although under basal conditions NPR-C specific agonist cANF does not affect HR, in the presence of a β-AR agonist, NPR-C signaling becomes important. Specifically, activation of NPR-C by cANF decreases AP firing in a dose-dependent manner in SAN myocytes that have been first stimulated with isoproterenol (Azer et al., 2012). This response is due to modulation of *I*_CaL_ by NPR-C/G_i_ activation causing a reduction in cAMP concentration in mouse SAN myocytes (Rose et al., 2004), although NPR-C activation does not modify *I*_f_ in any way (potentially due to compartmentalization of receptors and channels in the SAN). Interestingly, when β-agonist concentrations are high, activation of β-AR switches the actions of BNP and CNP from increasing HR to decreasing HR due to increasingly important contributions of NPR-C to the overall effects of NP (Moghtadaei et al., 2016).

In humans, the response to ANP has been variable. In some studies, ANP has had no effect on HR (Volpe et al., 1987, 1990; Crozier et al., 1993), while in others it has either increased or decreased HR in a dose-dependent manner (Biollaz et al., 1986; Bussien et al., 1986; Weidmann et al., 1986; Franco-Suarez et al., 1987; Nicholls and Richards, 1987). Although cellular and molecular mechanisms of ANP action have not been explored in the human SAN, in human atrial myocytes ANP has been shown to increase *I*_f_ and *I*_CaL_, likely *via* an increase in cytosolic cGMP and cAMP concentrations (Boixel et al., 2001; Lonardo et al., 2004). The influence, if any, of BNP or CNP on HR has not been shown in human, although they, along with ANP, are both found in the circulation and in the myocardium of humans (Moghtadaei et al., 2016).

So overall, NP plays an important role in controlling HR *via* specific modulations of pacemaker components in animal models, but how this translates to human remains uncertain. Responses are complicated because of the multiple receptors and the non-specificity of NPR binding by the various NP. Because endogenous levels of NP increase under certain pathological conditions, such as heart failure, it is important to understand the role they play in SAN function.

### Adenosine

Adenosine is a purine nucleoside formed from the breakdown of ATP that controls a plethora of cellular functions in an autocrine and paracrine fashion in the cardiovascular system, including in the SAN (Mangoni and Nargeot, 2008). Adenosine shares a similar signaling pathway to M_2_R and causes decreased HR in many species including human, dog, rabbit, guinea pig, rat, and mouse (Mangoni and Nargeot, 2008). Adenosine binds to purinergic receptors (A_1_, A_2A_, A_2B_, and A_3_), all of which are found in the heart (Headrick et al., 2013). A_1_ and A_3_ receptors are coupled to G_i_ and G_q/11_ pathways, causing a decrease in intracellular cAMP, whereas the A_2_ receptors are coupled to G_s_ and cause an increase (Headrick et al., 2013). However, it is the A_1_ receptor (A_1_R) that is attributed to modulating HR in the SAN (Figure 4). Adenosine application activates an inwardly rectifying K^+^ current (*I*_KAdo_) that is mediated by the same G-protein signaling and coupled K^+^ channels as *I*_KACh_ (Kir3.1/3.4) (Belardinelli et al., 1988). This has been demonstrated in the human isolated SAN where adenosine application decreases HR *via* A_1_R-induced GIRK channel activation, an effect that is abolished by GIRK channel blockade, implicating that the chronotropic response may be predominantly due to *I*_KAdo_ rather than alternative intracellular pathways and targets (Li et al., 2017). In humans there is greater expression of both A_1_R and GIRK4 in the SAN compared to the adjacent right atrium, suggesting the SAN might be more sensitive to adenosine than the surrounding tissue (Li et al., 2017). Interestingly, the sensitivity to adenosine-challenge is heart-specific, potentially explained by differences in A_1_R and GIRK protein expression between hearts. Although in humans GIRK channel activation is thought to be the main contributor to decreased HR, in animal models other mechanisms have been identified. Specifically, in rabbit SAN myocytes, adenosine also decreases HR through inhibition of *I*_CaL_ and *I*_f_, and causes a negative shift in the activation of *I*_f_, leading to a negative shift of MDP and a reduced SDD rate (West and Belardinelli, 1985; Belardinelli et al., 1988). Although activation of A_1_R by adenosine reduces HR, it has been shown that A_2A_ gene deletion in mice also reduces HR, implying that activation of A_2A_ would instead increase HR. A_3_ receptor deletion in mice, on the other hand, has the opposite effect, pointing to a role for A_3_ receptors in reduced HR, consistent with A_1_R (Headrick et al., 2013). And not only does adenosine contribute to normal daily rhythm control, it has also been implicated in pathophysiological settings. Upregulation of A_1_R has been demonstrated in dogs with induced chronic heart failure compared to control, which may worsen conduction abnormalities, leading to SAN dysfunction (Lou et al., 2014). Adenosine is produced in greater amounts in ischemia and heart failure, and can cause SAN conduction block and tachy-bradyarrhythmia in the dog (Lou et al., 2013).

### Angiotensin II

The renin-angiotensin system plays a critical role in cardiovascular and fluid homeostasis. Renin converts angiotensinogen to angiotensin I, which is then cleaved by angiotensin converting enzyme (ACE) into angiotensin II (ANGII), which is the main effector molecule (Mehta and Griendling, 2007). ANGII is an important and far reaching hormone, as it affects virtually all organs, including the heart, the kidneys, the vasculature, and the brain, yet knowledge of its modulatory effects on the SAN is limited and controversial (Mehta and Griendling, 2007; Sheng et al., 2011). Acutely, ANGII has been shown to decrease the firing rate of guinea pig SAN myocytes by a reduction in *I*_CaL_ and an increase in *I*_Ks_ (Sheng et al., 2011). Similarly, in rabbit SAN myocytes ANGII decreases spontaneous firing rate by decreasing *I*_CaL_ (Habuchi et al., 1995). However, in anesthetized dogs, ANGII injection increases HR (Lambert, 1995). This difference could be due to species-specific effects or related to differences in whole animal and single cell responses. ANGII acts on angiotensin (AT) receptors, AT_1_ and AT_2_, however, it is *via* AT_1_ that ANGII modulates the SAN (Sechi et al., 1992; Sheng et al., 2011). Signaling pathways downstream of AT_1_ are complex, and the mechanisms by which binding of ANGII to AT_1_ modulates automaticity is unclear. ANGII binding to AT_1_ can activate both G_q/11_ and G_i_, as well as G-protein independent pathways (Mehta and Griendling, 2007). AT_1_ activation also modulates other receptors in the plasma membrane and activates signaling molecules such as MAPK and arachidonic acid (Mehta and Griendling, 2007). Not only does ANGII act directly on myocytes, it can serve as a neuromodulator stimulating the release of catecholamines from sympathetic neurons (Marin-Garcia, 2011). Perhaps the multiple targets and complex signaling networks downstream of ANGII activation underlie the varying chronotropic effects seen in different experimental species and preparations. Interestingly, ANGII has not been shown to change HR in humans, despite its well documented role in regulation of blood pressure (Mehta and Griendling, 2007). Both AT receptors are present in the human heart, with higher AT_2_ expression in fetal development and in failing hearts compared to healthy adult hearts (Mehta and Griendling, 2007). Although no studies have been done in human tissue, when given ANGII intravenously no changes are observed in fetal or adult HR (Oney and Kaulhausen, 1982; Sander-Jensen et al., 1988). ANGII converting enzyme, however, is found in great quantity in the human SAN, perhaps indicating local synthesis of ANGII (Beaulieu and Lambert, 1998). ANGII is clearly a hormone that warrants further investigation regarding its role in HR control in human, especially as receptor expression and circulating ANGII concentration change substantially in pathology (Mehta and Griendling, 2007).

### Endothelin

Endothelin peptide hormones, ET-1, ET-2, and ET-3, are produced in vascular endothelial cells, and act as vasoconstrictors (Gordan et al., 2015). However, they have also been shown to affect automaticity of the SAN. Experimental studies have shown varying chronotropic effects following ET-1 application. ET-1 application to guinea pig right atrial tissue increases HR (Ishikawa et al., 1988), whereas decreased HR occurs in rabbit SAN myocytes (Tanaka et al., 1997; Ono et al., 2001). In anesthetized cats, ET-1 application results in increased HR at low doses but a biphasic response at higher doses, whereas ET-3 application only increases HR (Minkes et al., 1989). It has been proposed that these differing responses are due to differential activation of the two endothelin receptors A and B (ET_A_ and ET_B_). Both are expressed in the SAN, with ET_A_ causing a decrease and ET_B_ causing an increase in HR (Ono et al., 2001). ET_A_ activation by ET-1 hyperpolarizes membrane potential by activating *I*_KACh_, and inhibits *I*_CaL_ by way of G_i_ protein signaling (Ono et al., 2001). In pigs, intravenous infusion of an ET_A_ inhibitor causes an increase in HR, suggesting a role for endogenous ET and basal activation of ET_A_ receptors in the maintenance of HR (Gelzer et al., 2004). The pathway activated by ET_B_ in the SAN that results in an increase in HR requires further investigation. ET_B_ activation in endothelial cells causes NO production and activates the G_q_ protein cascade, so perhaps a similar pathway is activated in the SAN (Horinouchi et al., 2013). Minimal data exist regarding endothelin and its receptors in the SAN of human. In one study, patients with coronary artery disease were infused with the same ET_A_ inhibitor as in the pig study above, yet no change in HR was observed (Kolettis et al., 2003). Although this could relate to a species difference, it may also be a consequence of altered levels of basal ET and other neurohumoral agents affecting HR with coronary artery disease. It has also been shown that ET-1 levels contribute to the reduced HR variability seen in heart failure patients, perhaps by modulating SAN function (Aronson et al., 2001).

### Thyroid Hormones

Thyroid hormones are biogenic amines that are released from the thyroid gland and can influence HR over a longer time time scale than most agents (Mangoni and Nargeot, 2008). It is well documented in humans that high levels of thyroid hormone in the circulation (hyperthyroidism) lead to increased HR, whereas low levels of thyroid hormone (hypothyroidism) are associated with decreased HR. Thyroid hormones act on nuclear hormone receptors (thyroid receptors, TR) which are a group of intracellular ligand-dependent transcription factors of which there are two subtypes: TRα and TRβ (Mangoni and Nargeot, 2008). Their activation can alter the expression of different cardiac ion channels. In rabbit isolated SAN myocytes, incubation with triiodothyronine (T_3_) to mimic hyperthyroidism increases the amplitude of *I*_f_ without altering the voltage-dependence of the current (Renaudon et al., 2000). It has been shown in rats that T_3_ application significantly increases the mRNA expression level of HCN2 (Pachucki et al., 1999). Thus, it appears that an increase in *I*_f_ channel expression with T_3_ stimulation increases the density of I_f_, causing an increase in HR. In mouse lines meant to mimic hypothyroidism (TRα and TRβ KO), TRβ KO mice demonstrated no alterations in HCN expression level or HR, whereas the TRα KO had decreased HR and a reduction in both HCN2 and HCN4 expression levels, implying that the effects of T_3_ on HCN channel expression occur through activation of TRα (Gloss et al., 2001). Expression levels of both the Na^+^-Ca^2+^ exchanger and Na^+^/K^+^-ATPase have been shown to change based on thyroid concentration in rats, which could also affect HR (Mangoni and Nargeot, 2008). Thyroid hormones can also indirectly modulate HR in rats by increasing the number of β_1_-AR in the plasma membrane, increasing the ability for NE or epinephrine to increase HR (Dillmann, 1989). Thyroid hormones have also been shown to change HR in rats on a much shorter times scale, implicating a non-genomic mechanism perhaps mediated by eNOS (Rutigliano and Zucchi, 2017). Although there is no clear data describing specific mechanisms for the chronotropic effect of thyroid hormones seen in humans, it was shown that even when within a normal range in healthy individuals, there is a positive correlation between circulating T_3_ concentration and resting HR (Roef et al., 2013). In a study to determine the effect of thyroid hormones on the ANS control of HR, basal HR was higher in people with hyperthyroidism, and following complete ANS blockade, HR values remained higher in people with hyperthyroidism, suggesting the tachycardia with hyperthyroidism may at least in part be due to direct effect of thyroid hormones on the SAN (Valcavi et al., 1992). However, at least some component of the response is likely *via* indirect action through the ANS, as blockade of the sympathetic nerves alone reduces tachycardia in patients with hyperthyroidism (Valcavi et al., 1992). This could be due to the upregulation of β_1_-AR expression, as in rats. Although the intracellular mechanisms(s) are unclear, there is clearly a role for thyroid hormones in control of the human SAN. Understanding the specific mechanisms by which this happens is important for people with hyperthyroidism not only because of the determinantal effects of chronic tachycardia, but also because it increases the likelihood of atrial fibrillation, atrioventricular conduction abnormalities, and sinus node dysfunction (Valcavi et al., 1992).

### Parathyroid Hormone

Parathyroid hormone (PTH) is synthesized by the parathyroid gland and modulates many organs and tissues *via* the circulation in an endocrine fashion. PTH-related protein does not normally circulate in the blood but is present in most cells. It is produced in cardiomyocytes and acts locally as an autocrine or paracrine hormone (Shimoni, 1999). There are two parathyroid receptors, PTH1R and PTH2R, both of which increase intracellular cAMP concentrations (Potthoff et al., 2011). PTH1R is coupled to both the G_s_ and G_q_ protein pathways, thus increasing intracellular cAMP, PKA, PLC, IP_3_, and PKC (Chorev, 2002). PTH2R activation increases intracellular cAMP and activates receptor internalization (Usdin et al., 2002). In rabbit SAN myocytes, PTH increases AP firing frequency by increasing the amplitude of *I*_f_ and in turn the slope of SDD (Hara et al., 1997). PTH has also been shown to increase HR in rat isolated hearts (Shimoyama et al., 2001). In a clinical study involving chronic heart failure patients, there was a positive correlation between endogenous PTH levels and HR, suggesting a role for PTH in the associated increased HR (Sugimoto et al., 2013). Similarly, in two studies of patients undergoing kidney dialysis, increased endogenous PTH was associated with a decrease in HR variability, implicating PTH in the ANS dysfunction that occurs in individuals with kidney failure (Ussawawongaraya et al., 2013; Poulikakos et al., 2014). Although both PTH receptors are expressed in human atria and thus PTH might increase HR by directly affecting SAN myocytes as it does rabbit myocytes, it is also possible that observed increases in HR in humans are due to indirect effects on the SAN *via* the ANS (Potthoff et al., 2011). Specifically, in human atrial tissue PTH causes release of NE from sympathetic nerve terminals, which could account for an increase in HR, although this effect has not yet been demonstrated experimentally (Potthoff et al., 2011). Although it seems apparent that PTH modulates HR, there are still some areas for investigation, including understanding the role of PTH in HR control in healthy humans compared to pathological settings, the intracellular pathway(s) by which PTH affects human SAN myocytes and/or sympathetic nerve terminals, and the relative contribution of each mechanism to the chronotropic response with PTH.

### Bradykinin

Bradykinin is an inflammatory mediator peptide primarily known for its role in regulating blood pressure (Golias et al., 2007). Bradykinin receptors are GPCRs and have been reported to exist in the heart (Golias et al., 2007). With co-expression of one of these receptors (BK_2_R) and HCN1 or HCN2 in *Xenopus* oocytes, a modulatory effect of bradykinin on HCN channel dynamics has been demonstrated (Pian et al., 2007). BK_2_R is coupled to a G_q/11_ pathway that stimulates PLC activity, which causes a depolarizing shift in the activation of HCN channels (Pian et al., 2007). When bradykinin is applied to patch-clamped rabbit SAN myocytes, a similar depolarizing shift in *I*_f_ activation is observed (Pian et al., 2007), which would be expected to increase HR. This is supported by a study in anesthetized rats, in which intravenous bradykinin causes tachycardia (Ponchon et al., 1995). In contrast, however, BK_2_R knock-out mice have increased basal HR and bradykinin injections into the brainstem of the mouse decreases HR, suggesting that BK_2_R activation may in fact lower HR in mice (Madeddu et al., 1999). In patients both with and without atherosclerosis, bradykinin increases HR when injected into the left coronary artery, although no effect is seen when injected into the right coronary artery (Schaefer et al., 1996), which is a surprising result as the branch supplying blood to the SAN most commonly arises from the right coronary artery (Pejkovic et al., 2008). Clearly, our understanding of the role of bradykinin on HR regulation and SAN modulation is lacking, particularly in humans.

### Relaxin

Relaxin is a reproductive hormone that is released during the menstrual cycle and during pregnancy, with many known effects on the reproductive (Bell et al., 1987) and cardiovascular systems (Han et al., 1994a). Experimentally, relaxin has been shown to increase HR in rat atria and rabbit SAN myocytes (Kakouris et al., 1992; Han et al., 1994a). In rabbit SAN myocytes, relaxin increases firing rate by enhancing *I*_CaL_ in a cAMP/PKA dependent manner (Han et al., 1994a). Relaxin binds to GPCRs and increases intracellular cAMP, but the specific receptor subtypes and cascades in the SAN are unknown. Administration of relaxin has also been shown to have secondary therapeutic effects *via* inhibition of cardiac mast cell activation, preventing histamine release and subsequent tachyarrhythmias in the pig (Nistri et al., 2008). No studies have been reported specifically investigating the effects of relaxin on the SAN in humans, but it may be relevant in the context of cardiovascular complications that occur with pregnancy, as circulating relaxin levels are high during the first trimester (Bell et al., 1987). During this period there is also an increase in cardiac output (in preparation for the increased cardiac demand imposed from the development of the fetus) (Meah et al., 2016), so relaxin may play a role in increasing cardiac output during pregnancy (as has been shown in rats, through an increase in HR and contractility, Bell et al., 1987). Interestingly, relaxin can cross the placenta during the early stages of pregnancy, and circulating relaxin levels correlate with fetal HR, suggesting it may play a role in controlling HR of the developing fetus (Johnson et al., 1994). While it may be that relaxin’s influence on the SAN is an important normal control mechanism during pregnancy, in the absence of pregnancy it may have unwarranted effects on HR.

### Nitric Oxide

Nitric oxide (NO) is a gas produced in almost all cell types that has numerous cardiac effects (Massion et al., 2003). Even within the SAN itself, NO has diverse, and sometimes conflicting consequences, and is found as a component in many signaling pathways. NO production occurs in cardiomyocytes and in nearby endothelial cells, either by endothelial NO synthase (eNOS) or by neuronal NO synthase (nNOS), and in pathological situations NO can be produced by inducible NO synthase (iNOS) (Fischmeister et al., 2005). NO binds to soluble GC (sGC), which causes cGMP production, and therefore an increase in intracellular cGMP concentrations (Fischmeister et al., 2005). cGMP interacts with many targets to modulate HR, including PKG and PDEs. NO-activated cGMP attenuates *I*_CaL_ in rat and guinea pig SAN myocytes by PKG-mediated channel phosphorylation (Fischmeister et al., 2005). NO also has conflicting downstream effects on *I*_CaL_, as cGMP activates PDE2 (which should decrease *I*_CaL_), but also inhibits PDE3 (which should increase *I*_CaL_) (Fischmeister et al., 2005). Not all effects of NO are due to cGMP, however. It has been shown that low concentrations of NO can activate G_s_ proteins (which would be expected to increase HR), and NO can activate G_i_ proteins (which would be expected to decrease HR) (Fischmeister et al., 2005). It has also been shown that NO can activate *I*_f_ (Massion et al., 2003). An additional complicating factor is that NO modulates release of neurotransmitters from autonomic neurons. NO can inhibit the presynaptic release of NE from sympathetic neurons and facilitate the release of ACh from vagal neurons (Paterson, 2001).

Ultimately, it would seem that the multitude of ways in which NO can influence SAN automaticity makes it difficult to discern its net effect on HR (Chowdhary and Townend, 1999). Yet a series of human studies may have helped provide insight into its role in modulating HR (Chowdhary et al., 2000, 2002a,b, 2004). In healthy individuals, intravenous administration of a NOS inhibitor, as well as a control agent, caused an increase in blood pressure and a subsequent baroreflex-mediated decrease in HR; however, NOS caused less bradycardia than the control agent (Chowdhary et al., 2000). This indicates that inhibition of NOS removes the tonic excitatory effect of NO on vagal activity. Further, administration of exogenous NO and a control agent caused decreased blood pressure and a subsequent baroreflex-mediated increase in HR, yet the exogenous NO caused less tachycardia than the control agent, demonstrating the preservation of vagal control (Chowdhary et al., 2000). Although NO may also directly act on SAN myocytes, these experiments suggest that any such effects are overwhelmed by the effect of NO on the ANS. This was demonstrated in a study in human heart transplant recipients, whose hearts are disconnected from the ANS. In these patients, exogenous NO increased HR and NOS inhibition decreased HR, changes that were not a reflex response, as the control agents increased blood pressure but did not change HR (Chowdhary et al., 2002a). During heart failure, baroreflex-mediated parasympathetic activation is still dependent on NO synthesis (Chowdhary et al., 2002b), and stimulation of the endogenous NO pathway may be a strategy for increasing parasympathetic activity under conditions of adverse sympathetic overactivity, such as with ventricular arrhythmias (Chowdhary et al., 2004).

## Integrated Control of Neurohumoral Signaling

As there are a multitude of different neurohumoral factors that influence SAN activity, but share common intracellular signaling pathways, understanding their integration is important for understanding the combined effect on the SAN and HR.

### Intracellular Compartmentalization

Intracellular compartmentalization of the various complex signaling cascades in the SAN is essential for its proper function (Steinberg and Brunton, 2001). Not only are compartments physically formed in the membrane, such as caveolae and lipid rafts, compartmentalization also occurs by intracellular biochemical modulators. PDEs degrade cAMP and/or cGMP and the variety and abundance of PDEs helps modulate the cellular responses by controlling the duration and amplitude of cAMP/cGMP responses, both at basal levels and in response to neurohumoral stimulation (Zaccolo and Movsesian, 2007), as well as creating spatially and temporally distinct pools of cAMP and cGMP by forming functional diffusion barriers (Steinberg and Brunton, 2001; Yaniv et al., 2015). There are 8 different PDE families that have been identified in the heart: PDE1, PDE2, and PDE3 hydrolyze both cAMP and cGMP; PDE4, PDE7, and PDE8 are cAMP-specific enzymes; and PDE5 and PDE9 are cGMP-specific (Zaccolo and Movsesian, 2007). Not only do each of these PDE families have differential ability to enzymatically degrade cAMP and/or cGMP, they also have distinct effects in different cardiac cell types (Hua et al., 2012). In mouse isolated SAN myocytes, PDEs regulate basal firing rate, which is increased with global or selective PDE inhibition (PDE2, PDE3, or PDE4) (Hua et al., 2012). In addition, when PDE2 in mouse or human SAN myocytes, or PDE3 or PDE4 in mouse SAN myocytes is inhibited, *I*_CaL_ is activated at more negative membrane potentials and its current amplitude increases, accounting for the increase in firing rate (Rivet-Bastide et al., 1997; Hua et al., 2012). PDE3 also regulates *I*_Ks_ in guinea pig SAN myocytes *via* hydrolysis of cGMP, as its inhibition increases current amplitude (Shimizu et al., 2002).

Basal PDE degradation of cAMP in rabbit SAN myocytes has been shown to modulate and compartmentalize Ca^2+^-clock mechanisms such as RyR and PLB, as PDE inhibition increases the rate and amplitude of local Ca^2+^ releases (Vinogradova et al., 2008). This has been confirmed specifically for PDE1A in rabbit SAN myocytes, facilitated by its higher expression in the SAN compared to other regions of the heart (Lukyanenko et al., 2016). Although inhibition of single PDEs only moderately increases SAN myocyte firing, recently it has been shown that dual PDE3 and PDE4 inhibition synergistically increases basal SAN firing rate by ∼50% (Vinogradova et al., 2018a). Concurrent PDE3 and PDE4 activation modulates Ca^2+^-clock components, specifically *via* a decrease in cAMP and PKA phosphorylation, which suppresses local Ca^2+^ releases and decreases HR (Vinogradova et al., 2018b). Following blockade of RyR with ryanodine, dual PDE3 and PDE4 inhibition failed to increase spontaneous HR, implicating that modulation of the Ca^2+^-clock is necessary for basal synergistic effects of PDE3 and PDE4 on HR (Vinogradova et al., 2018b).

Not only do PDEs attenuate basal SAN firing, they also modulate the response to neurohumoral stimulation. Because so many of the neurohumoral pathways in the SAN activate AC and GC, without intracellular control these secondary messenger systems would be limited in their ability to specifically target a response (Fischmeister et al., 2006). In mouse SAN myocytes it has been shown that compartmentalization of HCN channels by PDEs may play an important role in regulating β_1_-AR modulation of *I*_f_ (St. Clair et al., 2017)_._ Specifically, it appears that PDE4 acts as a barrier that isolates HCN channels from the rest of the cell, so that under basal conditions they cannot be accessed by cAMP, and that PDE3 interacts with PKA to favor the β_1_-AR/cAMP/PKA/HCN pathway (rather than activation by direct binding of cAMP to HCN) (St. Clair et al., 2017).

Another important consideration in the SAN’s complex signaling network is crosstalk between cAMP and cGMP. Negative feedback pathways exist for both cAMP and cGMP, such that when they activate PKA or PKG, these kinases activate PDEs that then degrade cAMP or cGMP. Specifically, cAMP-dependent PKA stimulates PDE3 and PDE4, which degrades cAMP, while cGMP-stimulated PKG activates PDE5, thus inhibiting cGMP. These signals are also interconnected, as cGMP can activate or inactivate PDEs that degrade cAMP. Specifically, cGMP activates PDE2 (decreasing levels of cAMP) and inactivates PDE3 (increasing cAMP). More cGMP is required to activate PDE2 than to inhibit PDE3, thus the latter occurs more readily and at lower cGMP concentrations. Conversely, cAMP can inhibit cGMP production through PKA-activated PDE5. An important aspect of the above responses is that the particular PDE activated or inhibited by cGMP or cAMP partly depends on their concentration. This interplay and its importance during neurohumoral stimulation has been demonstrated in mouse SAN tissue, as following a β-AR-induced increase in firing rate, application of a PDE5 inhibitor decreases frequency, a response that is attenuated by PDE2 inhibition (Isidori et al., 2015). This suggests that by preventing degradation of cGMP by PDE5, more PDE2 is activated by cGMP, which then degrades cAMP and decreases HR.

Neurohumoral signaling pathways are not only controlled by PDE hydrolysis of cAMP and cGMP, but also by tethering of PKA to specific intracellular locations by A-kinase-anchoring proteins (AKAP) (Zaccolo and Movsesian, 2007). Sequestering of PKA into localized pools by anchoring proteins allows for targeted substrate phosphorylation. AKAP not only spatially restricts and controls PKA but can also temporally modulate the actions of PKA (Dodge-Kafka et al., 2006). Although the intracellular distribution and dynamics of cGMP are not as well understood as cAMP, it is likely that proteins analogous to AKAP localize the actions of PKG to specific regions (Dodge-Kafka et al., 2006). It has been shown that PKG directly anchors to NPR-A, and this compartmentalization of cGMP helps modulate responses elicited by activation from NP versus NO (Airhart et al., 2003). Specific control of PKA and PKG by anchoring proteins in the SAN, however, requires further investigation.

### Receptor Regulation

Regulators of G-protein signaling (RGS) catalyze the hydrolysis of G_α_-guanosine triphosphate (GTP) to G_α_-guanosine diphosphate (GDP), effectively inactivating the GPCR (Riddle et al., 2005). GTP to GDP hydrolysis is a rate-limiting step for signal termination, so the longer the G_α_ subunit is in its active form (G_α_-GTP) the longer a signal is activated in a cell (Mighiu and Heximer, 2012). RGS proteins inhibit G-protein signaling cascades by speeding up this intrinsically slow GTP hydrolysis.

Limited studies have been performed examining RGS proteins in the SAN, but it is clear that they are important for controlling the downstream effects of M_2_R activation in mouse SAN myocytes (Cifelli et al., 2008; Mighiu and Heximer, 2012; Stewart et al., 2012; Wydeven et al., 2014). RGS4 and RGS6 have been shown to be more abundantly expressed in the SAN than other cardiac tissues (Mighiu and Heximer, 2012; Wydeven et al., 2014). These endogenous RGS proteins oppose the decrease in HR following parasympathetic stimulation by accelerating GTPase activity to more rapidly terminate G protein signaling, thus impeding activation of hyperpolarizing GIRK channels (Berman et al., 1996). In cultured rabbit SAN cells, RGS2 overexpression has been shown to increase the phosphorylation of PLB along with HR, likely due to suppression of the G_i_ signaling cascade (Yang et al., 2012). Other RGS proteins are expressed in the heart, with important roles in controlling neurohumoral activation, but they have not been demonstrated specifically in SAN tissue or cells. For instance, in ventricular myocytes RGS2 proteins have been shown to be inhibitory downstream of catecholamines and ANGII activation by hydrolysis of β_2_-AR activated G_αi_ subunits, as well as α_1_-AR or AT activated G_αq_ subunits (Chakir et al., 2011).

G-protein coupled receptor kinases (GRK) contribute to β-AR desensitization. Seven characterized mammalian GRKs have been shown to be expressed in the heart, with GRK2 expression most prominent (Huang et al., 2014). When GPCRs are activated, GRK2 *trans*-locates from the cytosol to the plasma membrane by binding with the G_βγ_ subunit and phosphorylating the agonist-bound GPCR (Fu and Xiang, 2015). Following phosphorylation by GRK, the GPCR becomes a target for β-arrestin, which binds to the GRK-phosphorylated GPCR and physically inhibits its signaling cascade by sterically hindering interactions between the GPCR and the downstream G-proteins (a process called homologous desensitization) (Huang et al., 2014). Conversely, heterologous desensitization is initiated by PKA or PKC phosphorylation of the GPCR (Huang et al., 2014). GPCR phosphorylation increases the affinity of GPCR for adaptor proteins, which initiates clathrin-pit formation, which results in GPCR internalization (Fu and Xiang, 2015). Once internalized, the GPCR is then sorted to either recycling endosomes, to be trafficked back to the membrane, or to lysosomes for degradation (Fu and Xiang, 2015).

## Conclusion

One could argue that the SAN is one of the most important tissues in the body, as it initiates each-and-every normal heartbeat. Because the SAN is so critical to life, it is not surprising that it is heavily controlled, ensuring that the heart can adapt and respond to meet physiological demands. There are numerous overlapping and redundant mechanisms that drive automaticity in the SAN, to ensure that it continues to fire under widely varying conditions. The combination of these pacemaking mechanisms and their control by a multitude of neurohumoral factors creates an intricate and complex system (Li et al., 2017).

Despite a solid foundation, our current understanding of the complexities of the neurohumoral control of SAN activity is lacking. Further characterization of the distribution and expression of the many receptor types in SAN myocytes may help provide insight into the comparative contribution of the different neurohumoral agents. Often, the missing link in our current understanding of how a specific neurohumoral agent modulates HR lies between receptor activation and its intracellular target; i.e., we know what receptor an agonist activates and what pacemaker mechanisms it ultimately modulates, but the pathways involved are not clear. Also, in many cases investigations focus on effects on *I*_f_ and/or *I*_CaL_ and do not consider impacts on other important membrane currents such as *I*_Kr_ or *I*_Ks_, or on Ca^2+^-clock mechanisms, leaving us with an incomplete understanding of subcellular mechanisms driving observed HR responses.

There are many discrepancies in the literature regarding the effects of specific neurohumoral factors on HR. This may relate to species differences or differences in experimental approaches or preparations. At the level of the whole SAN, however, there may also be an important contribution of cellular heterogeneity. While we have focused on the intracellular modulation of SAN activity, it is important to appreciate that the SAN is structurally and electrophysiologically heterogeneous (Mangoni and Nargeot, 2008), so effects will vary across it. The SAN is heavily innervated, with a complex network of nerve fibers and ganglia whose density is much higher than the surrounding myocardium (Roberts et al., 1989; Chow et al., 2001; Newton et al., 2014; Pauza et al., 2014; Stoyek et al., 2015; Inokaitis et al., 2016). Yet the SAN is not ubiquitously innervated and this, along with variable receptor densities throughout the node, make it functionally heterogeneous (MacKaay et al., 1980; Opthof, 2007). Sympathetic control will dominate in heavily innervated regions and areas with high receptor density, whereas these regions may be silenced by parasympathetic control, such that areas with fewer receptors or little innervation would then determine rate. Moreover, neurohumoral stimulation can shift the location of the leading pacemaker site in the SAN, which itself can result in a change in HR, as different SAN myocytes have different intrinsic cycle lengths (Opthof et al., 1987).

Overall, it is clear that further work is needed for a complete, comprehensive understanding of the complex mechanisms involved in neurohumoral control of SAN function. This will be driven by technological advances in experimental techniques (Kohl and Quinn, 2014), such as higher resolution imaging and cell-specific optogenetics (Quinn et al., 2016), and may be further enhanced by the use of alternative experimental models (Stoyek et al., 2016, 2018; Stoyek and Quinn, 2018) or computational modeling (Quinn and Kohl, 2011, 2013). Ultimately, by improving our knowledge of SAN control we will move toward a better understanding of how it responds to constantly changing physiological demands, and how we may better treat SAN dysregulation that leads to debilitating and often deadly cardiac diseases.

## Author Contributions

EM designed the figures and wrote the manuscript. RR and TQ revised the manuscript. All authors approved the final version of the manuscript.

## Conflict of Interest

The authors declare that the research was conducted in the absence of any commercial or financial relationships that could be construed as a potential conflict of interest.
